# The role of TMEM119 in gastric adenocarcinoma and its specific effects on immunity

**DOI:** 10.1177/03000605241306668

**Published:** 2025-04-12

**Authors:** Yating Liu, Xin Yan, Caihao Qu, Futian Tang, Qian Wang, Yumin Li

**Affiliations:** 1 Department of Medical Oncology, Lanzhou University Second Hospital, Lanzhou, China; 2 Key Laboratory of Digestive System Tumors of Gansu Province, Lanzhou, China

**Keywords:** Stomach adenocarcinoma, TMEM119, prognosis, Tumor microenvironment, Immunity

## Abstract

**Objective:**

To investigate the prognostic significance and immunological implication of transmembrane protein 119 (TMEM119) in stomach adenocarcinoma (STAD).

**Methods:**

This study included STAD-associated data obtained from The Cancer Genome Atlas (TCGA) and Gene Expression Omnibus (GEO) databases. In addition, *TMEM119* expression levels were analysed by immunohistochemistry in tissue samples from patients with STAD (with microsatellite instability or microsatellite stability). Gene Set Enrichment Analysis (GSEA) was conducted to explore signalling pathways related to TMEM119 in STAD. Additionally, CIBERSORT and ESTIMATE algorithms were applied to examine the relationship between *TMEM119* expression and tumour-infiltrating immune cells, as well as the tumour microenvironment.

**Results:**

Surgical specimens from 100 patients with STAD (50 each with microsatellite stability or microsatellite instability); TCGA RNA-sequence and clinical data from 375 STAD tumour tissues and 32 paracancerous tissues; and two GEO datasets (GSE27342, comprising 80 paracancerous tissues and 80 tumour tissues; and GSE84437, comprising 433 tumour samples) were analysed. TMEM119 was found to be elevated in STAD, and associated with poor prognosis. Clinical gastric cancer tissues exhibited increased *TMEM119* expression. *TMEM119* was enriched in immune-related functions and pathways. *TMEM119* correlated with immune checkpoint genes, tumour mutational burden, and microsatellite instability. TMEM119 was positively correlated with tumour-infiltrating immune cells, tumour microenvironment, mannose receptor C-type I (CD206), and programmed cell death-ligand 1 (PD-L1), while inversely related to nitric oxide synthase 2.

**Conclusions:**

TMEM119 may be a potential immune-related biomarker for STAD prognosis and therapeutic targeting.

## Introduction

Stomach adenocarcinoma (STAD) is a predominant malignancy, characterized by rapid progression and high rates of metastasis,^1^ and the prognosis of patients with advanced-stage STAD remains poor. The 5-year survival rate for patients with early STAD may surpass 90%, while the 5-year survival rate is below 30% for patients with advanced STAD.^2^ For most patients with STAD, the efficacy of conventional treatments is limited. Therefore, it is vital to explore novel therapeutic targets that might help to improve the effectiveness of immunotherapy.

Immune-checkpoint inhibitors have become the leading immunotherapy approach in the treatment of STAD.^3^ Programmed cell death 1 (PDCD1, also known as PD-1) and programmed cell death-ligand 1 (PD-L1) inhibitors decrease the size of tumours and prolong the overall survival of a subset of patients with gastric cancer.^4,5^ Highly unstable microsatellites and mismatch repair defects are important prognostic indicators for tumours. Currently, patients with STAD who harbour these indicators are sensitive to treatment with immune-checkpoint inhibitors and the objective response rate is about 50%.^6^ Although these results have led to the approval of such treatment as a promising therapy for patients with gastric adenocarcinoma that is positive for mismatch repair defects/highly unstable microsatellites, about half of the patients remain unresponsive.

Tumour mutation burden, tumour immunosuppressive microenvironment, and *PD-L1* expression are strongly associated with the response to immune-checkpoint inhibitors.^7,8^ In addition, the advantages of these inhibitors in improving prognostic outcomes in STAD has been shown to vary with different patient ethnicities, different immune-checkpoint inhibitor categories, microsatellite instability, and tumour mutational burden.^9^ Furthermore, many genetic biomarkers currently ignore clinical pathological features and do not reflect the immune status of patients. Thus, it is crucial to investigate immune response factors and look for new immunotherapeutic targets for STAD.

Transmembrane protein 119 (TMEM119), also known as an osteogenic inductive factor, belongs to the transmembrane protein family, and early research has revealed that TMEM119 is essential for normal bone mineralization and bone growth.^10^ In addition to its functions in osteogenesis, previous studies reported that TMEM119 contributes to tumorigenesis, such as osteosarcoma, ovarian cancer, and breast cancer.^11–13^ In addition, TMEM119 deficiency has been shown to result in the apoptosis of gastric carcinoma cells,^14^ and *TMEM119* overexpression was shown to aggravate the invasion and migration of gastric tumour cells.^15^ However, the effects of TMEM119 on the tumour microenvironment, particularly the tumorous immune microenvironment, remain poorly described. Thus, the aim of the present study was to investigate the role of TMEM119 in the tumorous immune microenvironment of STAD and uncover the underlying mechanism, by processing the data of patients with STAD derived from The Cancer Genome Atlas (TCGA) database and analysing tissue samples from patients with STAD with microsatellite instability or stability. Through investigating the clinical prognosis and immunological implications of TMEM119 in STAD, the relationship between TMEM119, prognosis and tumour microenvironment was revealed.

## Materials and methods

### Surgical samples and ethics

Paraffin-embedded surgical specimens (tumour tissues and adjacent histopathological sections) from 100 consecutive patients with STAD (50 with microsatellite stability and 50 with microsatellite instability confirmed by pathology) were acquired from the Department of Pathology at the Lanzhou University Second Hospital. The study was approved by the ethics committee of Lanzhou University Second Hospital (No. 2022 A-637) and all patients provided written informed consent. The study was conducted according to the 1975 Declaration of Helsinki as revised in 2013.

### Samples from online databases

RNA-sequence data comprising 375 STAD tumour tissues and 32 paracancerous tissues, and their corresponding clinical data, were downloaded from the TCGA database (https://portal.gdc.cancer.gov/). In addition, the following STAD datasets were obtained from the Gene Expression Omnibus (GEO) database (https://www.ncbi.nlm.nih.gov/geo/): GSE27342, comprising 80 paracancerous tissues and 80 tumour tissues, and GSE84437, comprising 433 tumour samples. The gene expression matrix was processed via the ‘limma’ R language package.

### Gene expression analysis

*TMEM119* expression in STAD and other cancers was analysed using the Tumor Immune Estimation Resource (TIMER) online platform (https://cistrome.shinyapps.io/timer/), a useful instrument that incorporates several algorithms to unveil the expression levels of genes in cancer and normal specimens.^16^ *TMEM119* expression levels in the TCGA-STAD and GSE27342 datasets were analysed through the ‘limma’ R package.

#### Correlation analysis of TMEM119 with clinicopathological and prognostic factors

The ‘survminer’ and ‘survival’ packages in R (https://cran.r-project.org/web/packages/survminer; https://cran.r-project.org/web/packages/survival) were used to analyse the correlation of TMEM119 with clinicopathological and prognostic factors in TCGA-STAD patients. Cox regression analysis was performed to calculate the hazard ratio (HR) and 95% confidence intervals.

#### Functional enrichment analysis

Genes that were co-expressed with *TMEM119* were analysed in the TCGA-STAD RNA Seq data by Pearson's correlation coefficient. Differentially expressed genes (DEGs) between groups with high or low *TMEM119* expression were filtered out using |LogFC| > 1 and FDR < 0.05 (where FC = fold change; FDR = false discovery rate) as a standard. Gene Ontology (GO) and Kyoto Encyclopedia of Genes and Genomes (KEGG) enrichment analysis were carried out to investigate the pathways of the DEGs. TMEM119-related pathways in STAD were predicted using gene set enrichment analysis (GSEA) software, version 4.3.2 (https://www.gsea-msigdb.org/gsea/index.jsp).

#### Tumour microenvironment analysis

Levels of immune cell infiltration and immune function activation were quantified using the single sample GSEA algorithm. Tumour microenvironment scores were calculated for each patient with STAD from the TCGA database, with the R package ‘ESTIMATE’ (Estimation of STromal and Immune cells in MAlignant Tumor tissues using Expression data; https://bioconductor.org/packages/release/bioc/html/estimate.html), mainly used to evaluate the proportion of stromal and immune cells in the tumour microenvironment. The abundance of tumour-infiltrating immune cells in STAD samples was estimated by the CIBERSORT software package (https://cybersort.stanford.edu).

#### Evaluation of sensitivity to chemotherapeutic agents

To determine the sensitivity to chemotherapy of STAD patients, the ‘pRRophetic’ R language package (https://github.com/paulgeeleher/pRRophetic), which predicts chemotherapeutic response using tumour gene expression data, was used to assess the half-maximum inhibitory concentration (IC_50_) of chemotherapeutic drugs.

#### Immunohistochemistry

Paraffin-embedded tumour and adjacent histopathological tissue sections from patients with STAD were deparaffinized and rehydrated, and then antigen recovery was performed in a citric acid tissue antigen repair solution (MVS-0101; MXB, Fuzhou, China). The sections were incubated with the following primary antibodies: rabbit anti-TMEM119 (1:200 dilution, AMAb91528; Atlas Antibodies, Stockholm, Sweden), rabbit anti-nitric oxide synthase 2 (NOS_2_; 1:100 dilution, ab115819; Abcam, Cambridge, UK), rabbit anti-ICDC206 (1:100 dilution, ab252921; Abcam), and rabbit anti-PD-L1 (1:100 dilution, ab205921; Abcam), overnight at 4°C after blocking with UltraSensitive™ SP solutions (KIT-9710; MXB). After washing, sections were then incubated with horse-radish peroxidase-conjugated secondary antibody for 1 h at room temperature. Immunoreactions were visualized by 3,3′-diaminobenzidine (DAB) solution (DAB-0031; MXB). The intensity of staining and the percentage of positive cells were scored separately by two pathologists: no staining, 0; light yellow, 1; brown yellow, 2; and tan, 3. The percentage of positive cells ≤ 5% was 0 points, 6–25% was 1 point, 26–50% was 2 points, 51–75% was 3 points, and ≥ 76% was 4 points. The two items were multiplied for the final score, with < 6 defined as low expression and ≥ 6 as high expression.

#### Statistical analyses

Statistical analyses and graphical visualization were performed using R software, version 4.1.3 (https://cran.r-project.org/src/base/R-4/R-4.1.3.tar.gz). Wilcoxon rank-sum test was used to analyse differences in gene expression. The association between TMEM119 and clinical features was evaluated by χ^2^-test. A *P* value < 0.05 was deemed statistically significant.

## Results

### Levels of TMEM119 expression in STAD

**Figure 1.**
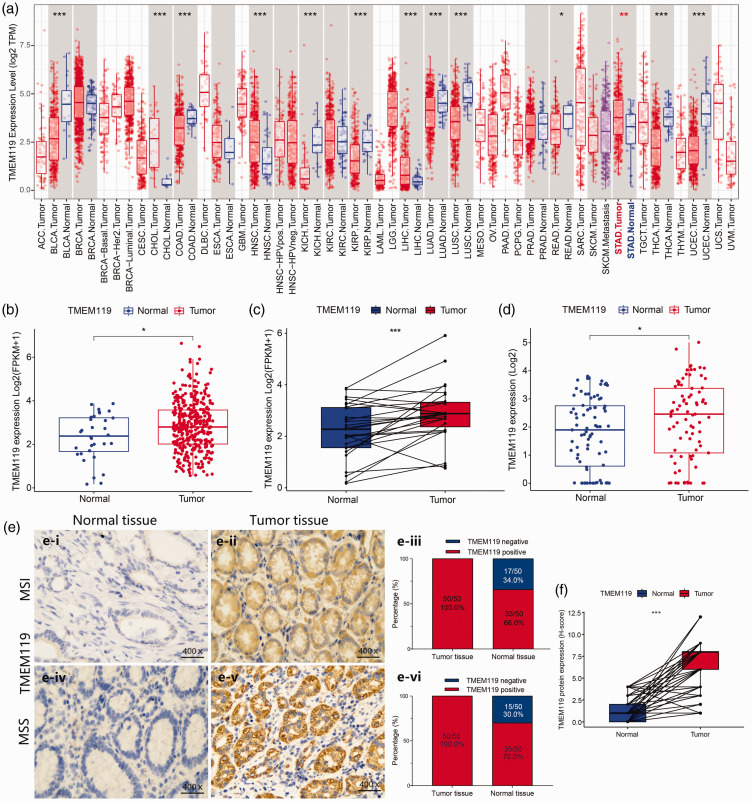
Analysis of transmembrane protein 119 (*TMEM119*) expression in normal tissues and cancer tissues, including stomach adenocarcinoma (STAD), showing: (a) box-whisker plot of *TMEM119* expression levels in tumour and normal tissue from different cancers using the TIMER database; (b) *TMEM119* expression levels in STAD unpaired samples from The Cancer Genome Atlas (TCGA) database; (c) *TMEM119* expression levels in STAD samples from TCGA database; (d) *TMEM119* expression levels in 80 normal tissues and 80 tumour tissues from the Gene Expression Omnibus GSE27342 dataset; (e-i, ii, iv and v) representative immunohistochemistry images of TMEM119 staining in normal or tumour tissue samples from 100 patients with STAD with microsatellite instability (MSI) or microsatellite stability (MSS); and (e-iii, vi, and f) statistical analyses of these samples. *P <0.05, **P < 0.01, ***P < 0.001.

Using the TIMER database, TMEM119 was found to be upregulated in STAD, as well as in other cancers, such as hepatocellular carcinoma, head and neck squamous cell carcinoma, and cholangiocarcinoma (Figure 1(a); *P* < 0.05). Further investigation of *TMEM119* expression in STAD samples from the TCGA database revealed that TMEM119 was upregulated in tumour versus normal tissues, in both unpaired and paired-sample analyses (Figure 1(b) and (c); *P* < 0.05). In addition, analysis of data from the GSE27342 dataset confirmed this result (Figure 1(d); *P* < 0.05).

Expression of *TMEM119* at the protein level in tumour and adjacent normal tissue from 100 STAD clinical samples (50 with microsatellite instability and 50 with microsatellite stability) was investigated using immunohistochemistry, and revealed that the TMEM119 protein was primarily localized on the cell membrane (Figure 1e-i, ii, iv and v). In 50 samples with microsatellite instability, all of the tumour tissues showed a high level of TMEM119 expression, and only 66% of normal tissues produced TMEM119 (Figure 1e–iii). A similar result was shown in 50 samples with microsatellite stability, which revealed TMEM119 was positive in all STAD tumour tissues and 70% of normal tissues (Figure 1e–iv). Furthermore, H-scores for immunohistochemical staining were significantly higher in STAD tumour tissues compared with adjacent normal tissues (Figure 1(f); *P* < 0.001).

### Association between TMEM119 and clinicopathology factors

**Figure 2.**
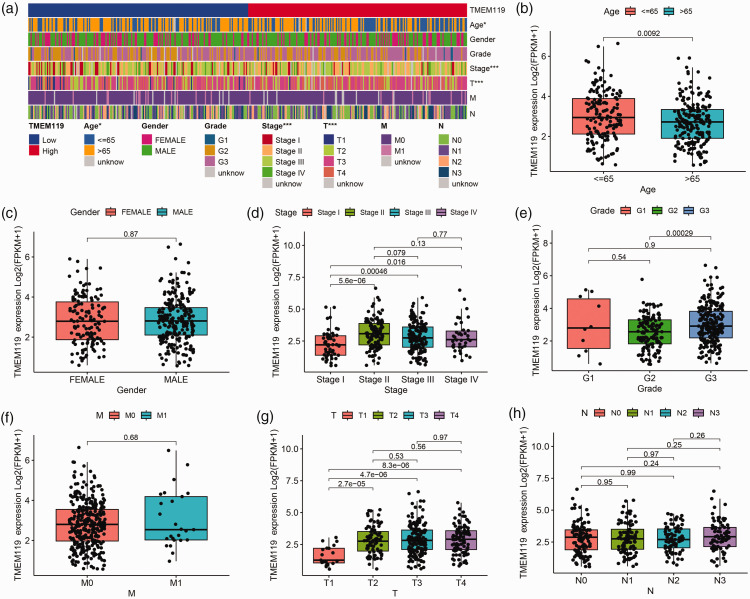
Analysis of the relationship between transmembrane protein 119 (*TMEM119*) expression and clinicopathological features in stomach adenocarcinoma (STAD) data from The Cancer Genome Atlas (TCGA) database, showing: (a) heat map of *TMEM119* expression in a different age, genders, histological grade, clinical stage, M stage, T stage, and N stage; and the (b–h) The association of TMEM119 with (b) age, (c) gender, (d) clinical stage, (e) histological grade, (f) M stage, (g) T stage, and (h) N stage. *P <0.05, **P < 0.01, ***P < 0.001 (χ^2^-test).

The relationship between TMEM119 and clinical factors was explored in STAD (Figure 2(a)). The results revealed that *TMEM119* expression was significantly associated with age (*P* = 0.0092; Figure 2(b)), cancer stage (*P* < 0.05; Figure 2(d)), grade (*P* = 0.00029; Figure 2(e)), and T stage (*P* < 0.0001; Figure 2(g)). However, there were no associations between TMEM119 and gender (Figure 2(c)), M stage (Figure 2(f)), or N stage (Figure 2(h)).

### The prognostic value of TMEM119 in STAD

**Figure 3.**
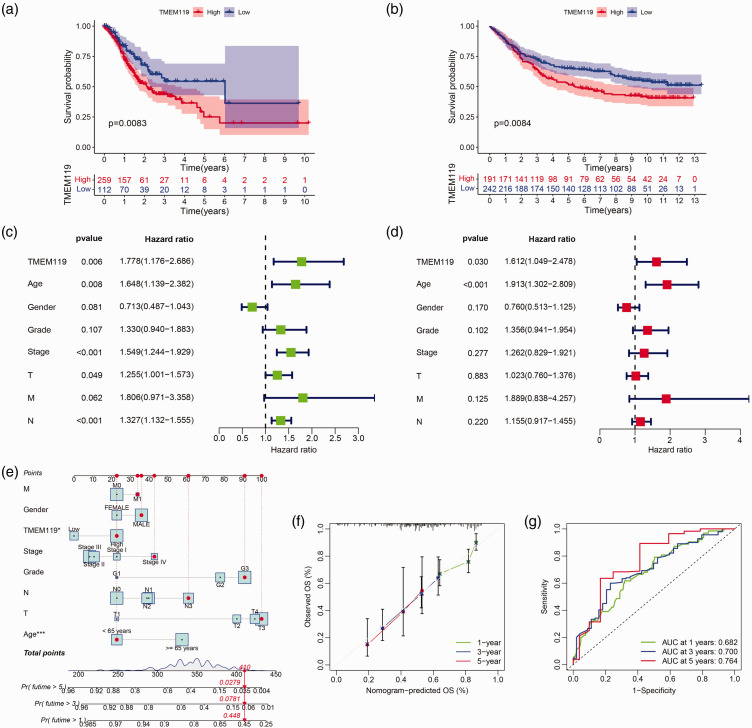
Analysis of the prognostic effect of transmembrane protein 119 (*TMEM119*) in stomach adenocarcinoma (STAD): Kaplan-Meier survival curves in patients with STAD grouped according to high or low TMEM119 from (a) The Cancer Genome Atlas (TCGA) and (b) GSE84437 datasets; (c) univariate and (d) multivariate Cox regression analysis of TMEM119 and clinicopathological factors in STAD-TCGA data showing hazard ratios and 95% confidence intervals; (e) a nomogram constructed based on TMEM119; (f) calibration curve showing correlation between observed overall survival (OS) and nomogram-predicted OS; and (g) receiver-operating characteristic curves with area under the curve values for 1-, 3-, and 5-year overall survival.

The prognostic value of TMEM119 in STAD was explored. Kaplan-Meier survival curves revealed that higher levels of *TMEM119* expression were associated with worse overall survival in patients with STAD from both TCGA-STAD and GSE84437 datasets (Figures 3(a) and (b)). In addition, TMEM119 was shown to be an independent prognostic determinant in STAD by univariate and multivariate Cox regression (Figure 3(c) and (d)). Further, a nomogram model that incorporated clinicopathological variables and TMEM119 was constructed (Figure 3(e)), and revealed that there was good uniformity between the nomogram-predicted overall survival and observed overall survival at 1, 3, and 5 years for STAD (Figure 3(f)). Area under the curve calculations (using receiver operating characteristic curves) for 1-, 3-, and 5-year overall survival were 0.682, 0.700, and 0.764, respectively (Figure 3(g)).

### GO and KEGG enrichment analysis

To further understand the function of TMEM119 in STAD, genes that were co-expressed with TMEM119 in STAD were analysed using Pearson’s correlation coefficient. Expression of serpin family F member 1 (*SERPINF1*), raftlin, lipid raft linker 1 (*RFTN1*), synapse defective Rho GTPase homolog 1 (*SYDE1*), V-set and transmembrane domain containing 4 (*VSTM4*), and zinc finger E-box binding homeobox 2 (*ZEB2*) were positively correlated with TMEM119. Other genes, such as small nuclear ribonucleoprotein polypeptide G (*SNRPG*), ubiquitin C-terminal hydrolase L5 (*UCHL5*), small nucleolar RNA host gene 1 (*SNHG1*), and ubiquitin conjugating enzyme E2 T (*UBE2T*) were found to be inversely correlated with TMEM119 (**Supplementary Figure 1a and b**). Next, the dominant functions and pathways related to these DEGs were investigated by employing GO and KEGG. GO enrichment analysis revealed that the functions of DEGs were focused on leukocyte migration, positive regulation of chemotaxis, and T cell receptor complex (**Supplementary Figure 1c**). The main associated pathways of DEGs manifested by KEGG analysis were transforming growth factor (TGF)-β and Wnt signalling pathways (**Supplementary Figure 1d**). After categorising the DEGs according to high or low *TMEM119* expression, the pathways of DEGs related to high *TMEM119* expression were revealed to be observably enriched in immune-related pathways, such as Janus kinase (JAK)/signal transducers and activators of transcription (STAT), B/T cell receptor, and TGF-β signalling pathways (**Supplementary Figure 1e**).

### Tumour microenvironment and TMEM119 analysis in STAD

**Figure 4.**
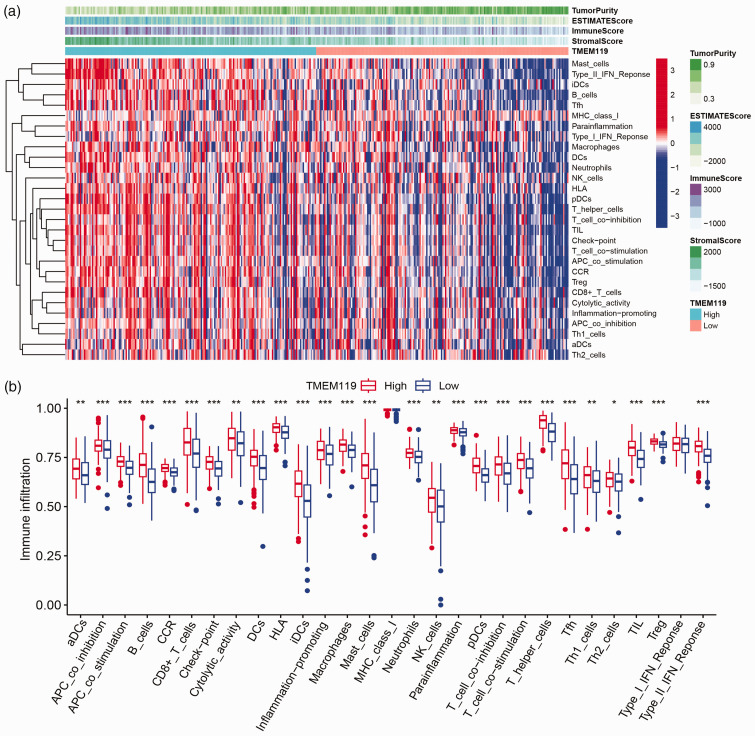
Single sample gene set enrichment analysis of the relationship between tumour microenvironment and transmembrane protein 119 (TMEM119) in stomach adenocarcinoma (STAD)-The Cancer Genome Atlas (TCGA) data, showing: (a) heatmap of immune cells and tumour microenvironment score; and (b) differences in immune cell infiltration levels in the high and low TMEM119 groups analysed using Wilcoxon rank-sum test. *P < 0.05, **P < 0.01, ***P < 0.001.

To explore the relationship between tumour microenvironment and TMEM119, the immune cell infiltration levels and immune cell types, and immune activation, were analysed by ssGSEA algorithm. The results indicated that immune cell infiltration (including macrophages and T helper cells) and levels of immune function (including human leucocyte antigen, inflammation-promoting, and type I/II interferon responses) were higher in patients with high levels of *TMEM119* expression than in those with low levels (Figure 4(a) and (b)). In addition, the ImmuneScore, StromalScore, and ESTIMATEScore were determined in STAD samples with the ESTIMATE algorithm. Patients with high levels of *TMEM119* expression were found to have higher ESTIMATEScore, ImmuneScore, and StromalScore in the tumour microenvironment (**Supplementary Figure 2a, d and g**). Furthermore, Kaplan Meier survival analysis was performed and showed that patients with high StromalScore or high ESTIMATEScore had a poor prognosis (**Supplementary Figure 2b, e and h**). Interestingly, patients with a high StromalScore or ESTIMATEScore combined with high TMEM119 were found to have the worst prognosis (**Supplementary Figure 2c, f and i**).

**Figure 5.**
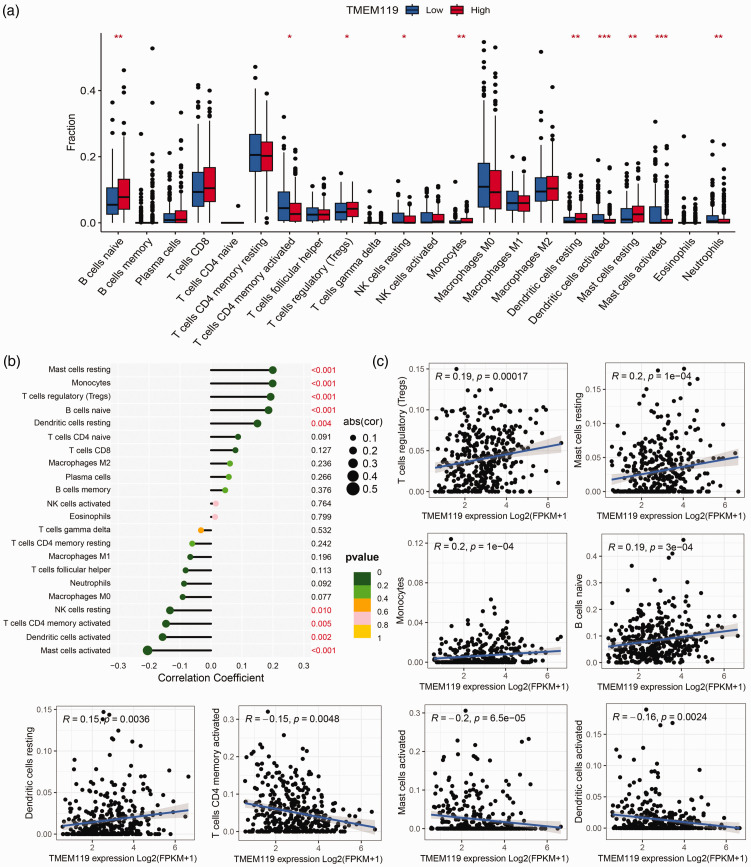
Analysis of the relationship between tumour-infiltrating immune cells and transmembrane protein 119 (TMEM119) in TCGA-STAD RNA-Seg data, showing: (a) differences in tumour-infiltrating immune cell content between tissues with high versus low *TMEM119* expression; and (b and c) correlation between tumour-infiltrating immune cells and *TMEM119* expression levels. FPKM, Fragments Per Kilobase of transcript per Million mapped reads. *P < 0.05, **P < 0.01, ***P < 0.001 (Wilcoxon signed-rank test and Spearman’s rank correlation coefficient).

The immune cell composition infiltrating STAD tumour tissue (out of 22 tumour-infiltrating immune cell types) was investigated in patients with STAD using the CIBERSORT algorithm. Subsequent statistical analysis, performed using Wilcoxon signed-rank test, identified that naïve B cells, regulatory T cells, and monocytes were more abundant in patients with high *TMEM119* expression levels (*P* < 0.05; Figure 5(a)). Correlation analysis found that TMEM119 was positively correlated with naïve B cells, regulatory T cells and monocytes, but inversely associated with dendritic cells, mast cells, and CD4 T cells (Figure 5(b) and (c)).

### TMEM119 association with immune checkpoint genes, tumour mutational burden and microsatellite instability

**Figure 6.**
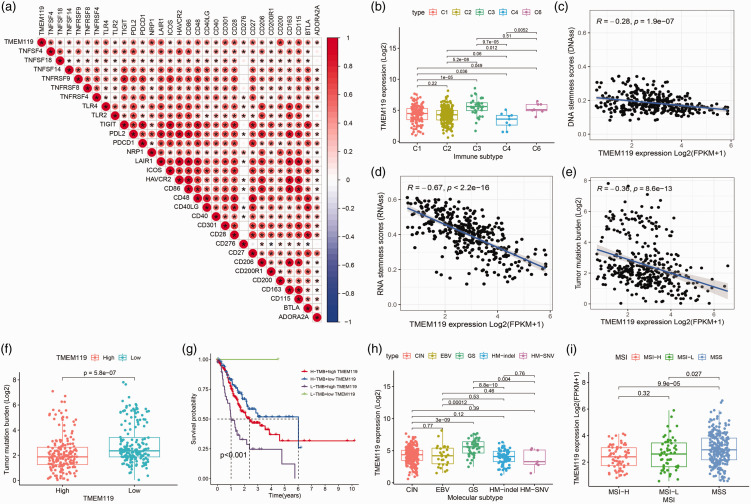
Analysis of the relationship between transmembrane protein 119 (TMEM119) and immune checkpoint genes, tumour mutational burden, and microsatellite instability, showing: (a) correlation between immune checkpoint genes and TMEM119 (Pearson’s correlation test); (b) *TMEM119* expression in samples grouped according to immune subtype; (c) correlation between DNAss and *TMEM119* expression; (d) correlation between RNAss and *TMEM119* expression; (e) correlation between tumour mutational burden and *TMEM119* expression; (f) levels of tumour mutational burden in groups with high or low *TMEM119* expression; (g) survival analysis in samples grouped according to high/low tumour mutational burden and high/low *TMEM119* expression; (h) *TMEM119* expression according to molecular subtype; and (i) TMEM119 expression according to microsatellite instability level (high, low or microsatellite instability). CIN, chromosomal instability; EBV, Epstein–Barr virus; FPKM, Fragments Per Kilobase of transcript per Million mapped reads; GS, genomically stable; HM-indel, hypermutated insertions/deletions; HM-SNV, hypermutated single-nucleotide variant; MSI, microsatellite instability; MSS, microsatellite stability; TMB, tumour mutational burden.

During this study, TMEM119 was found to be closely related to tumour microenvironment. To examine the associations between TMEM119 and immune checkpoint molecules in STAD, the expression of immune checkpoint genes (*PDCD1*, neuropilin 1 [*NRP1*], toll like receptor 4 [*TLR4*], and hepatitis A virus cellular receptor 2 [*HAVCR2*]) and *TMEM119* were analysed by Peason’s correlation coefficient. The results indicated that the majority of immune checkpoint genes were significantly positively correlated with TMEM119 (Figure 6(a)). Furthermore, the level of *TMEM119* expression was compared between five immune subtypes (STAD without C5 subtype: C1 – Wound Healing; C2 – IFN-γ Dominant; C3 – Inflammatory; C4 – Lymphocyte Depleted; C6 – TGF-β Dominant) and revealed differing levels of *TMEM119* expression between the various immune subtypes (Figure 6(b)). To quantify cancer stemness, the RNA stemness score (RNAss) and DNA stemness score (DNAss) were assessed, and revealed that *TMEM119* expression was significantly inversely correlated with DNAss and RNAss (r = –0.28 and r = –0.67, respectively, *P* < 0.0001; Figure 6(c) and (d)). Regarding tumour mutational burden in STAD, *TMEM119* expression was found to be significantly inversely associated with tumour mutational burden (r = –0.36, *P* < 0.0001; Figure 6(e)), and the tumour mutational burden score was higher in the low *TMEM119* expression group (Figure 6(f)). Further survival analysis showed that patients with STAD with low tumour mutational burden and high TMEM119 expression had the worst prognosis (Figure 6(g)). In addition, STAD contains four molecular subtypes: Epstein–Barr virus (EBV), genomically stable (GS), hypermutated single-nucleotide variant (HM-SNV), chromosomal instability (CIN), and microsatellite instability. Analysis of the correlation showed differences in *TMEM119* expression between the four molecular subtypes (Figure 6(h)). Subsequent analysis of the correlation between *TMEM119* expression and microsatellite instability showed that *TMEM119* expression was inversely associated with microsatellite instability (Figure 6(i)).

### Efficacy analysis of antitumor drugs

To assess the relationship between *TMEM119* expression and drug sensitivity, the IC_50_ of chemotherapeutic agents was analysed via pharmacogenomics. The results showed that patients with low *TMEM119* expression might be more sensitive to chemotherapeutic agents such as 5-fluorouracil, gemcitabine, and etoposide (**Supplementary Figure 3a–c**), whereas patients with high *TMEM119* expression levels might be more sensitive to chemotherapeutic agents such as sunitinib, pazopanib, and rapamycin (**Supplementary Figure 3d–f**).

### Immunohistochemical analysis of NOS2, CD206, PD-L1 and TMEM119

**Figure 7.**
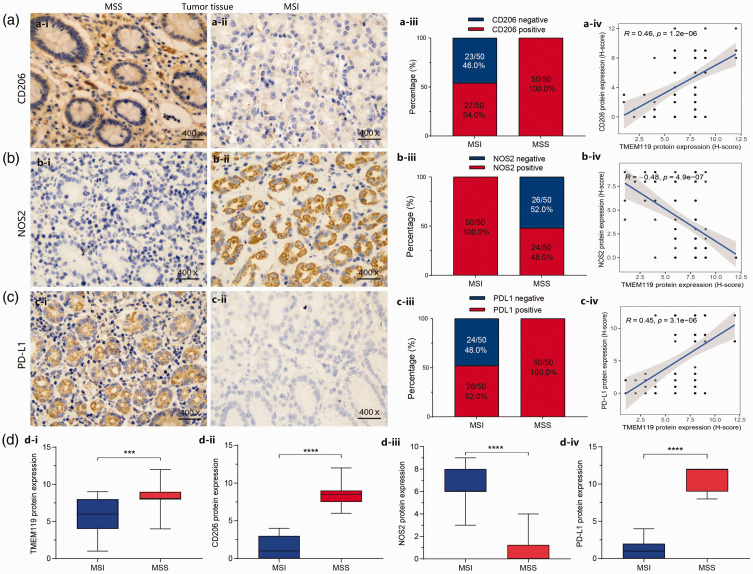
Immunohistochemical analysis of mannose receptor C-type I (CD206), nitric oxide synthase 2 (NOS2), programmed cell death-ligand 1 (PD-L1), and transmembrane protein 119 (TMEM119) protein levels in tumour tissues from 100 patients with stomach adenocarcinoma, grouped according to microsatellite instability (MSI, *n = *50) or stability (MSS, *n* = 50), showing: representative protein immunostaining images, % positive protein staining, and correlation analyses for (a) CD206, (b) NOS2, and (c) PD-L1; and (d) analysis of TMEM119, CD206, NOS2, and PD-L1 protein levels according to MSI (*n* = 50) or MSS (*n* = 50). *P < 0.05, **P < 0.01, ***P < 0.001, ****P < 0.0001 (Spearman’s rank correlation coefficient).

To understand whether TMEM119 may be related to the M2 polarization of macrophages, leading to poor prognosis, the protein levels of NOS2 (an M1 macrophage marker), CD206 (an M2 macrophage marker), PD-L1 and TMEM119 were analysed by immunohistochemistry in STAD tissues (50 samples with microsatellite instability and 50 samples with microsatellite stability). The results revealed that the CD206 protein was mainly localized in the cytoplasm (Figure 7a-i and ii), and its expression was positively correlated with the level of TMEM119 (r = 0.46, *P* < 0.0001; Figure 7a-iv). NOS2 protein was mostly distributed in the cytoplasm (Figure 7b-i and ii) and was inversely associated with TMEM119 (r = –0.48, *P* < 0.0001; Figure 7b-iv). PD-L1 protein was mainly localized in the cytoplasm (Figure 7c-i and ii) and was positively correlated with TMEM119 (r = 0.45, *P* < 0.0001; Figure 7c-iv). The proportion of patients with STAD exhibiting positive protein results for CD206, and PD-L1 were higher in 50 samples with microsatellite stability than in 50 samples with microsatellite instability (Figure 7a-iii and c-iii), while the level of NOS2 showed the opposite result (Figure 7b-iii). In addition, TMEM119-positive immunostaining was analysed in the present tissue samples from 100 cases of STAD, and showed that TMEM119 protein levels were low in 38 cases and high in 62 cases. Analysis of H scores for protein levels revealed that levels of TMEM119, CD206 and PD-L1 were significantly higher in samples with microsatellite stability versus instability (*P < *0.001; Figure 7d-i, ii and iv), and NOS2 protein levels were lower in those with microsatellite stability (*P* < 0.0001; Figure 7d-iii). The association between TMEM119 protein level (high or low) and clinicopathological features was analysed in 100 patients with STAD (**Supplementary Table 1**). Higher proportions of patients with high TMEM119 protein levels (H-score ≥ 6) were found in those aged <65 years (versus ≥ 65 years; *P* = 0.0278)) and in those with microsatellite stability versus instability (*P* = 0.0002). No other statistically significant associations were observed (regarding gender, TNM stage, depth of invasion, or lymph-node metastasis. The results suggest that TMEM119 may be significantly correlated with age and microsatellite instability.

## Discussion

The tumour microenvironment has been shown to have a significant impact on the effectiveness of tumour immunotherapy.^17^ Cancer cells are an important mediator of the immune tolerance of tumours and the functions of tumour-infiltrating immune cells, which would influence the clinical efficacy of immunotherapy,^18^ however, regulation of the tumour microenvironment, particularly the tumorous immune microenvironment in STAD, remains poorly understood. The present study explored the immune implication of TMEM119 in STAD, and revealed that elevated *TMEM119* expression in STAD correlates with poor patient prognosis and is closely linked to the tumour microenvironment. Therefore, TMEM119 may have the potential to serve as a new biomarker for diagnosis, prognosis, and targeted therapy, and might surpass traditional clinical markers, such as carcinoembryonic antigen (CEA), carbohydrate antigen (CA)72-4.

The current study revealed a novel immune-related factor, TMEM119, which may act as a potential therapeutic target of STAD. Previous research has reported that TMEM119 is an important factor in tumorigenesis. For example, TMEM119 was shown to accelerate the proliferation of ovarian cancer cells via the phosphatidylinositol 3-kinase (PI3K)/AKT signalling pathway,^12^ and Wnt/β-catenin signalling was shown to be involved in the positive role of TMEM119 in breast cancer.^13^ In addition, TMEM119 exhibited a tumour-promoting effect against gastric cancer through activation of STAT3 signalling.^15^ TMEM119 deficiency may lead to apoptosis of gastric adenocarcinoma cells via BCL2 associated X, apoptosis regulator (BAX)/caspase-3.^14^ In line with these studies, the present findings revealed that TMEM119 might serve as a biomarker for the molecular and pathological diagnosis of STAD. Additionally, Cox regression analysis and nomograms unveiled that TMEM119 was an independent prognostic indicator of STAD. In addition, the JAK-STAT, TGF-β, and Wnt signalling pathways were shown to be underlying pathways associated with the function of TMEM119 in STAD development. Due to the possible differences between bioinformatics and reality, it remains necessary to confirm these mechanisms of TMEM119 in STAD in future studies.

The tumour microenvironment serves as an important marker for predicting clinical outcomes and immunotherapy responsiveness,^19^ and the efficacy of immunotherapy has been shown to depend on the accumulation and activity of tumour-infiltrating immune cells.^20^ Tumour-associated macrophages have been reported to elevate angiogenesis in gastric cancer, possibly by upregulating vascular endothelial growth factor production.^21^ The recruitment of pro-inflammatory tumour-associated macrophages was suggested to be a new independent prognostic element for gastric cancer.^22^ Furthermore, more CD8^+^ cell infiltration has been inversely correlated with less angiogenesis in gastric cancer.^14^ Interestingly, the current study found that *TMEM119* expression might affect tumour microenvironment scores, immune cell infiltration, and immune function of STAD, implying that TMEM119 may be crucial for the regulation of immune function and immune cell recruitment in STAD.

Tumour mutational burden and microsatellite instability are regarded as predictive biomarkers for immunotherapy.^23–25^ In addition, a previous study revealed that patients with gastric cancer and higher tumour mutational burden scores had better survival outcomes.^26^ In the present study, TMEM119 was found to be significantly inversely associated with tumour mutational burden and patients with low tumour mutational burden and high TMEM119 had a poor prognosis, consistent with the abovementioned previous reports. STAD has been divided into four molecular subtypes in TCGA: microsatellite instability, EBV, GS, and CIN,^27^ with microsatellite instability having a higher rate of mutation and more immunotherapeutic potential.^28,29^ Meta-analysis revealed that patients with gastric cancer with microsatellite instability have a higher response rate to PD-1/PD-L1 inhibitors than patients with microsatellite stability.^9,30^ A previous study showed that alteration in the balance of M1/M2 macrophage polarization may lead to different immune characteristics of tumours.^31^ Thus, drug-induced polarization of tumour-associated macrophages from M2 to M1 phenotype may be a new strategy for treating tumours.^32^ In the present study, *TMEM119* expression was shown to be higher in samples with microsatellite stability than microsatellite instability. Given this result, tissue samples were obtained from patients with STAD with microsatellite instability or stability (50 cases each) and levels of NOS2 (an M1 macrophage marker), CD206 (an M2 macrophage marker), and PD-L1 were detected by immunohistochemistry. The results showed that TMEM119 was closely related to CD206, NOS2, PD-L1, and microsatellite instability in STAD. In addition, protein levels of TMEM119, CD206, and PD-L1 were higher in tissues exhibiting microsatellite stability versus instability, while NOS2 levels showed the opposite results. Taken together, the present results suggest that TMEM119 may act on macrophage polarization and satellite instability, resulting in immune escape in STAD. Therefore, it may be hypothesised that reducing *TMEM119* expression may improve the immune response to immune-checkpoint inhibitors in patients with STAD and high TMEM119 levels. Thus, the present research provides a new idea for immune-checkpoint-inhibitor treatment.

Overall, the present study substantially elucidates the role of TMEM119 in the tumour microenvironment in STAD, providing novel insights into its evaluation as a biological prognostic biomarker and immunotherapeutic target. However, the present results may also be limited by several factors. For example, the immune role of TMEM119 in the tumour microenvironment of STAD was analysed and predicted mainly using bioinformatics methods, and was not validated. Therefore, the results need to be confirmed by subsequent in-depth studies.

## Conclusions

In summary, the present study showed that *TMEM19* expression was upregulated in STAD, and TMEM119 played an influential role in immune cell infiltration and immune regulation. In addition, the research revealed that TMEM119 was positively related to the expression of *CD206*, a classical marker of M2 macrophages. The results provide novel ideas and theoretical support for TMEM119 as a novel potential prognostic indicator and immunotherapeutic target for STAD.

## Supplemental Material

sj-pdf-1-imr-10.1177_03000605241306668 - Supplemental material for The role of TMEM119 in gastric adenocarcinoma and its specific effects on immunity Supplemental material, sj-pdf-1-imr-10.1177_03000605241306668 for The role of TMEM119 in gastric adenocarcinoma and its specific effects on immunity by Yating Liu, Xin Yan, Caihao Qu, Futian Tang, Qian Wang and Yumin Li in Journal of International Medical Research

sj-pdf-2-imr-10.1177_03000605241306668 - Supplemental material for The role of TMEM119 in gastric adenocarcinoma and its specific effects on immunity Supplemental material, sj-pdf-2-imr-10.1177_03000605241306668 for The role of TMEM119 in gastric adenocarcinoma and its specific effects on immunity by Yating Liu, Xin Yan, Caihao Qu, Futian Tang, Qian Wang and Yumin Li in Journal of International Medical Research

sj-pdf-3-imr-10.1177_03000605241306668 - Supplemental material for The role of TMEM119 in gastric adenocarcinoma and its specific effects on immunity Supplemental material, sj-pdf-3-imr-10.1177_03000605241306668 for The role of TMEM119 in gastric adenocarcinoma and its specific effects on immunity by Yating Liu, Xin Yan, Caihao Qu, Futian Tang, Qian Wang and Yumin Li in Journal of International Medical Research

sj-pdf-4-imr-10.1177_03000605241306668 - Supplemental material for The role of TMEM119 in gastric adenocarcinoma and its specific effects on immunity Supplemental material, sj-pdf-4-imr-10.1177_03000605241306668 for The role of TMEM119 in gastric adenocarcinoma and its specific effects on immunity by Yating Liu, Xin Yan, Caihao Qu, Futian Tang, Qian Wang and Yumin Li in Journal of International Medical Research

## Data Availability

All data generated or analysed during this study are included in this published article.
