# Supplementary figures and images for: The role of TMEM119 in gastric adenocarcinoma and its specific effects on immunity
Source: J Int Med Res. 2025 Apr 12;53(4):03000605241306668. doi: 10.1177/03000605241306668 (PMC12033527; doi:10.1177/03000605241306668)

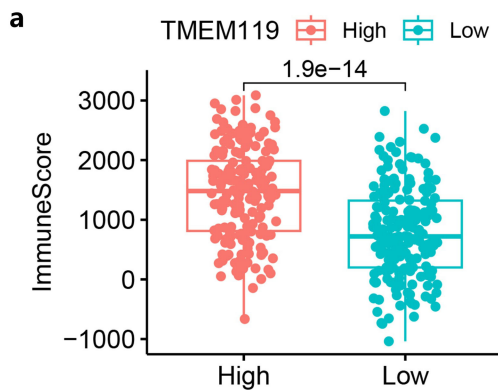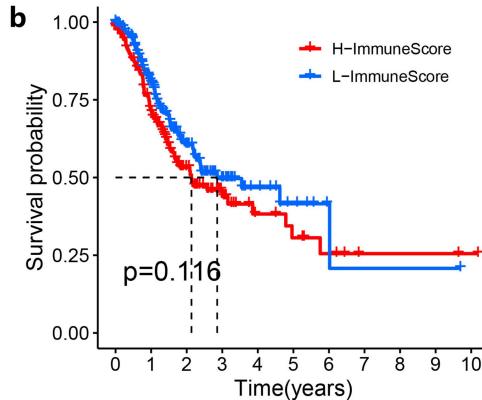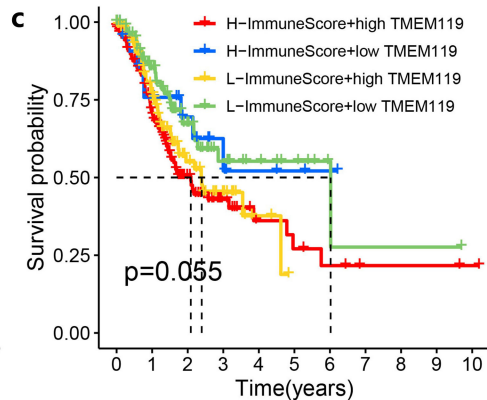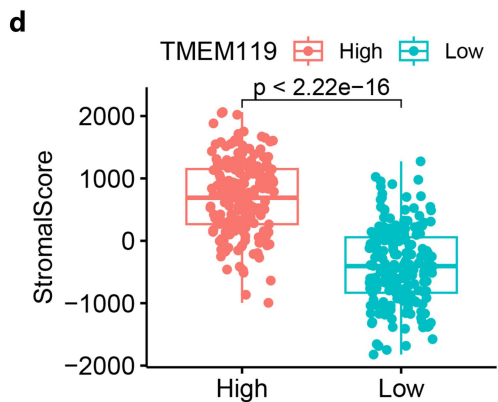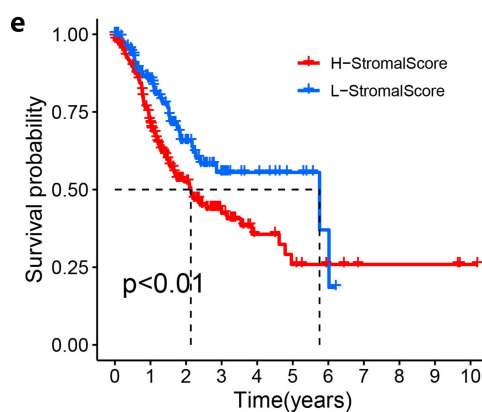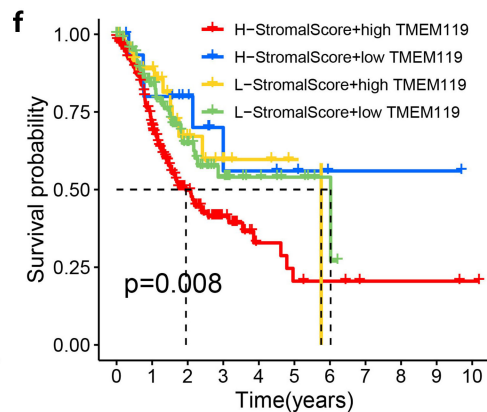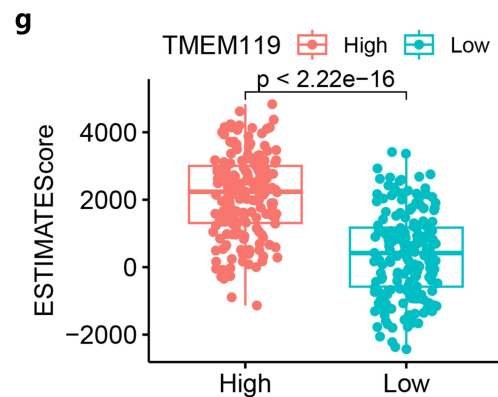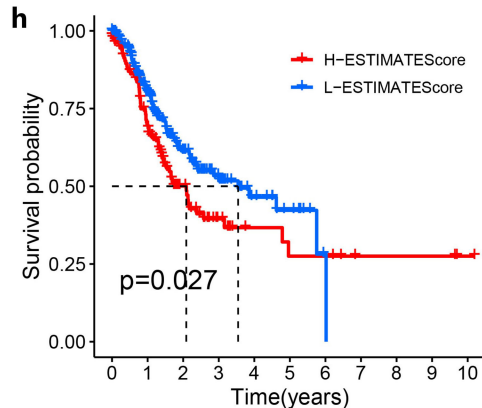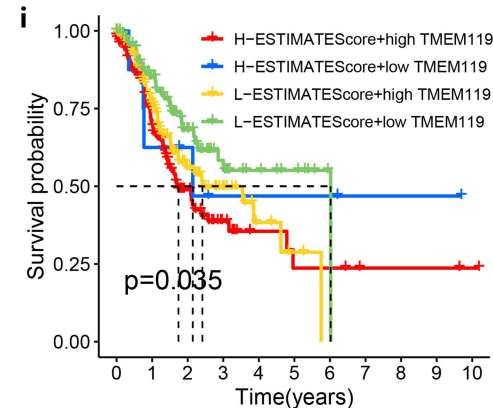

Supplement: sj-pdf-2-imr-10.1177_03000605241306668 - Supplemental material for The role of TMEM119 in gastric adenocarcinoma and its specific effects on immunity [file sj-pdf-2-imr-10.1177_03000605241306668.pdf]

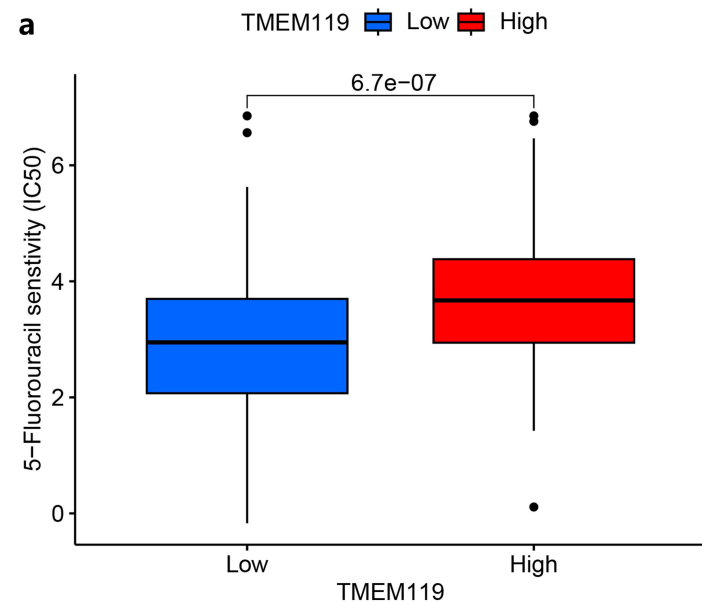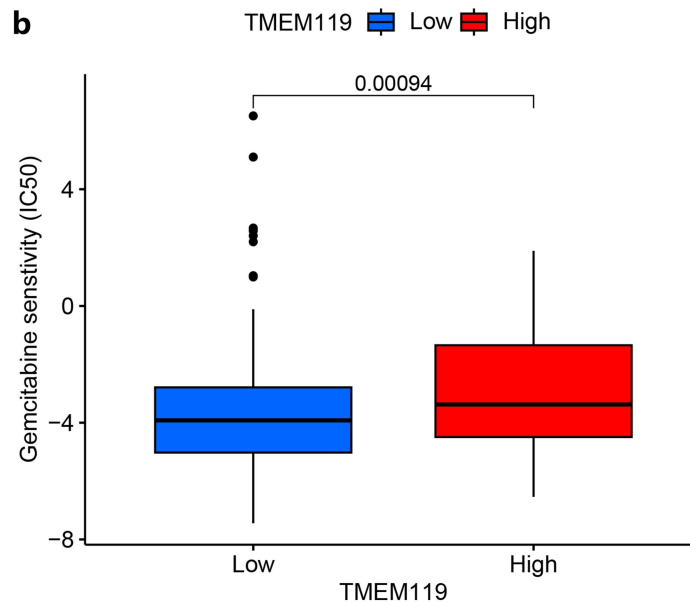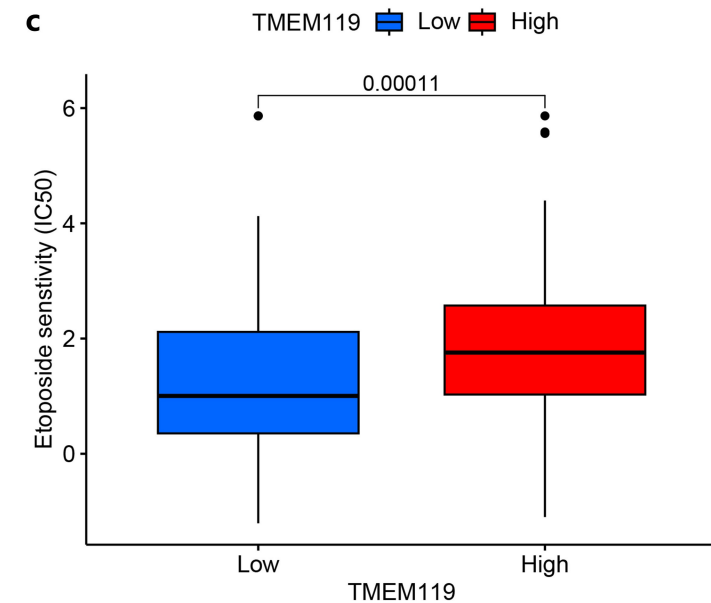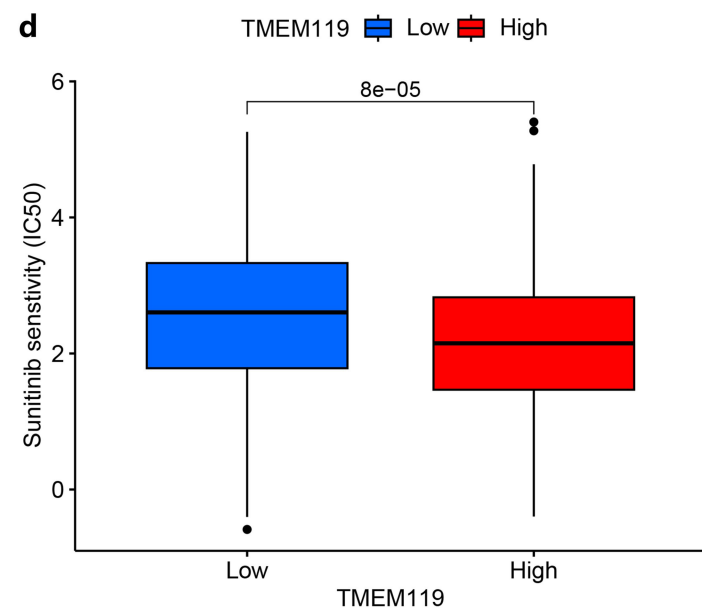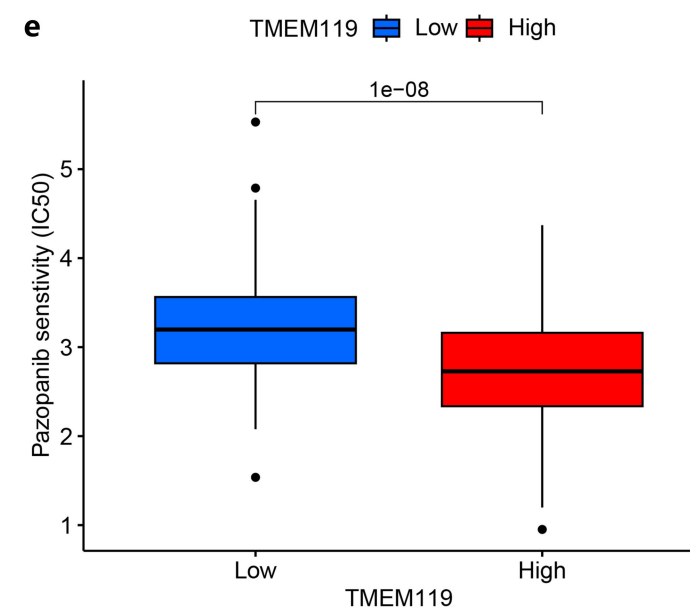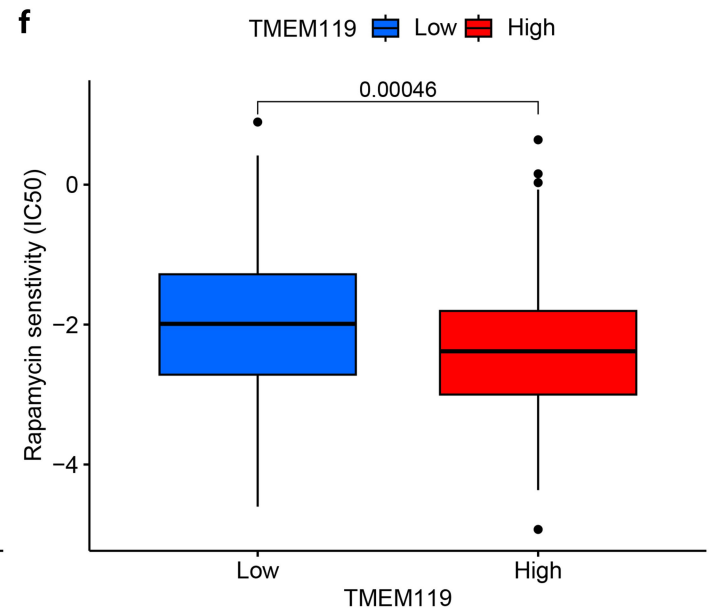

Supplement: sj-pdf-3-imr-10.1177_03000605241306668 - Supplemental material for The role of TMEM119 in gastric adenocarcinoma and its specific effects on immunity [file sj-pdf-3-imr-10.1177_03000605241306668.pdf]
