# Supplementary material for: The role of TMEM119 in gastric adenocarcinoma and its specific effects on immunity
Source: J Int Med Res. 2025 Apr 12;53(4):03000605241306668. doi: 10.1177/03000605241306668 (PMC12033527; doi:10.1177/03000605241306668)
Supplement: sj-pdf-4-imr-10.1177_03000605241306668 - Supplemental material for The role of TMEM119 in gastric adenocarcinoma and its specific effects on immunity [file sj-pdf-4-imr-10.1177_03000605241306668.pdf]

Table 1. Association between TMEM119 and clinicopathological features of STAD

Clinical characteristics	Total (n)	TMEM119 protein expression		χ^2	p-value
		High	Low		
**Age**				4.838	**0.0278**
< 65	73	50 (68.5%)	23 (31.5%)		
≥65	27	12 (44.4%)	15 (55.6%)		
**Gender**				1.173	0.2789
Male	72	47 (65.3%)	25 (34.7%)		
Female	28	15 (53.6%)	13 (46.4%)		
**TNM stage**				0.8532	0.3556
I+II	54	32 (59.3%)	22 (40.7%)		
III+IV	46	30 (65.2%)	16 (34.8%)		
**Depth of invasion (T)**				0.6751	0.8790
T1	17	12 (70.6%)	5 (29.4%)		
T2	21	13 (61.9%)	8 (38.1%)		
T3	15	9 (60.0%)	6 (40.0%)		
T4	47	28 (59.6%)	19 (40.4%)		
**Lymph-node metastasis (N)**				1.398	0.7060
N0	49	31 (63.3%)	18 (36.7%)		
N1	10	5 (50.0%)	5 (50.0%)		
N2	21	12 (57.1%)	9 (42.9%)		
N3	20	14 (70.0%)	6 (30.0%)		
**Lauren**				1.775	0.4117
Diffuse	37	24 (64.9%)	13 (35.1%)		
Intestinal	39	21 (53.8%)	18 (46.2%)		
Mixed	23	16 (69.6%)	7 (30.4%)		
**MSI/MSS**				13.75	**0.0002**
MSI	50	22 (44.0%)	28 (56.0%)		
MSS	50	40 (80.0%)	10 (20.0%)		
