# Supplementary material for: The role of TMEM119 in gastric adenocarcinoma and its specific effects on immunity
Source: J Int Med Res. 2025 Apr 12;53(4):03000605241306668. doi: 10.1177/03000605241306668 (PMC12033527; doi:10.1177/03000605241306668)
Supplement: sj-pdf-1-imr-10.1177_03000605241306668 - Supplemental material for The role of TMEM119 in gastric adenocarcinoma and its specific effects on immunity [file sj-pdf-1-imr-10.1177_03000605241306668.pdf]

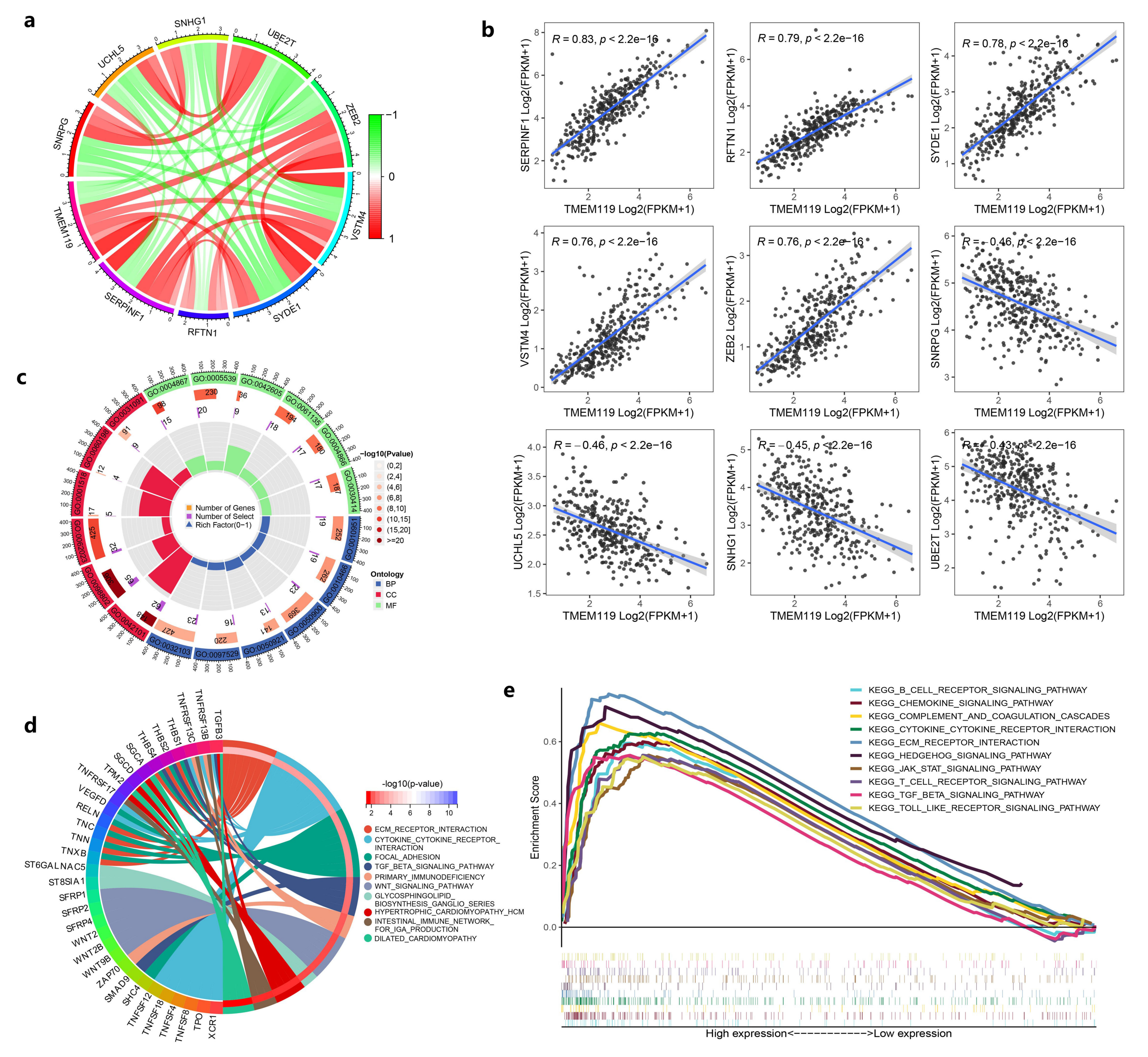

a
b
c
d
e
SNHG1
UBE2T
ZEB2
VSTM4
SYDE1
RFTN1
SERPINF1
TMEM119
SNRPG
UCHL5
R = 0.83, p < 2.2e-16
R = 0.79, p < 2.2e-16
R = 0.78, p < 2.2e-16
R = 0.76, p < 2.2e-16
R = 0.76, p < 2.2e-16
R = −0.46, p < 2.2e-16
R = −0.46, p < 2.2e-16
R = −0.45, p < 2.2e-16
R = −0.43, p < 2.2e-16
SERPINF1 Log2(FPKM+1)
RFTN1 Log2(FPKM+1)
SYDE1 Log2(FPKM+1)
VSTM4 Log2(FPKM+1)
ZEB2 Log2(FPKM+1)
SNRPG Log2(FPKM+1)
UCHL5 Log2(FPKM+1)
SNHG1 Log2(FPKM+1)
UBE2T Log2(FPKM+1)
TMEM119 Log2(FPKM+1)
Number of Genes
Number of Select
Rich Factor(0−1)
-log10(Pvalue)
(0,2]
(2,4]
(4,6]
(6,8]
(8,10]
(10,15]
(15,20]
>=20
Ontology
BP
CC
MF
GO:0005539
GO:0042605
GO:0061135
GO:0004866
GO:0030414
GO:0010951
GO:0010466
GO:0050900
GO:0050921
GO:0097529
GO:0032103
GO:0042101
GO:0098802
GO:0062023
GO:0001518
GO:0030198
GO:0031091
GO:0004867
-log10(p-value)
ECM_RECEPTOR_INTERACTION
CYTOKINE_CYTOKINE_RECEPTOR_INTERACTION
FOCAL_ADHESION
TGF_BETA_SIGNALING_PATHWAY
PRIMARY_IMMUNODEFICIENCY
WNT_SIGNALING_PATHWAY
GLYCOSPHINGOLIPID_BIOSYNTHESIS_GANGLIO_SERIES
HYPERTROPHIC_CARDIOMYOPATHY_HCM
INTESTINAL_IMMUNE_NETWORK_FOR_IGA_PRODUCTION
DILATED_CARDIOMYOPATHY
TGFB3
TNFRSF13B
TNFRSF13C
THBS1
THBS2
THBS4
SGCA
SGCD
TPM2
TNFRSF17
VEGFD
RELN
TNC
TNN
TNXB
ST6GALNAC5
ST8SIA1
SFRP1
SFRP2
SFRP4
WNT2
WNT2B
WNT9B
ZAP70
SMAD9
SHC4
TNFSF12
TNFSF18
TNFSF4
TNFSF8
TPO
XCR1
KEGG_B_CELL_RECEPTOR_SIGNALING_PATHWAY
KEGG_CHEMOKINE_SIGNALING_PATHWAY
KEGG_COMPLEMENT_AND_COAGULATION_CASCADES
KEGG_CYTOKINE_CYTOKINE_RECEPTOR_INTERACTION
KEGG_ECM_RECEPTOR_INTERACTION
KEGG_HEDGEHOG_SIGNALING_PATHWAY
KEGG_JAK_STAT_SIGNALING_PATHWAY
KEGG_T_CELL_RECEPTOR_SIGNALING_PATHWAY
KEGG_TGF_BETA_SIGNALING_PATHWAY
KEGG_TOLL_LIKE_RECEPTOR_SIGNALING_PATHWAY
Enrichment Score
High expression<------------>Low expression
